# Heavy Metal and Trace Element Status and Dietary Determinants in Children with Phenylketonuria

**DOI:** 10.3390/nu16203463

**Published:** 2024-10-12

**Authors:** İzzet Erdal, Yılmaz Yıldız, Siddika Songül Yalçın, Anıl Yirün, Deniz Arca Çakır, Pınar Erkekoğlu

**Affiliations:** 1 Clinic of Pediatric Metabolic Diseases, Etlik City Hospital, 06170 Ankara, Türkiye; 2 Department of Social Pediatrics, Institute of Child Health, Hacettepe University, 06230 Ankara, Türkiye; siyalcin@hacettepe.edu.tr; 3 Division of Pediatric Metabolism, Department of Pediatrics, Hacettepe University İhsan Doğramacı Children’s Hospital, 06230 Ankara, Türkiye; yilmaz.yildiz@hacettepe.edu.tr; 4 Division of Social Pediatrics, Department of Pediatrics, Hacettepe University İhsan Doğramacı Children’s Hospital, 06230 Ankara, Türkiye; 5 Department of Vaccine Technology, Vaccine Institute, Hacettepe University, 06230 Ankara, Türkiye; arcideniz@gmail.com (D.A.Ç.); erkekp@hacettepe.edu.tr (P.E.); 6 Department of Pharmaceutical Toxicology, Faculty of Pharmacy, Çukurova University, 01330 Adana, Türkiye; anil.yirun@gmail.com; 7 Department of Pharmaceutical Toxicology, Faculty of Pharmacy, Hacettepe University, 06230 Ankara, Türkiye

**Keywords:** heavy metals, trace elements, phenylketonuria, phenylalanine-restricted diet, toxicity

## Abstract

**Background/Objectives:** Heavy metals are a group of metals and metalloids that have a relatively high density. They can cause toxicity even at very low levels. Trace elements are required by all living organisms to maintain their normal growth, metabolism, and development. Oral intake is the main route of exposure to both heavy metals and trace elements. Phenylketonuria (PKU) is the most common amino acid metabolic disorder, and the best known treatment for patients requiring treatment is a phenylalanine (Phe)-restricted diet. The objective of the present study was to evaluate the plasma heavy metal levels, sources of exposure, changes in these levels according to dietary regimen, and trace element levels and their correlations with heavy metals in PKU patients. **Methods:** The study was conducted between July 2022 and January 2024 on 105 patients aged 2–6 years diagnosed with PKU. **Results:** The percentage of Pb levels in individuals in the upper quartile increased by 3.47 times (95% CI = 1.07–11.29) in those who consumed canned foods and 7.29 times (95% CI = 1.21–44.03) in those who consumed spring water. The percentage of As levels in the upper tertile increased by a factor of 7.26 (95% CI = 2.09–25.28) in individuals under four years of age and 8.17 times (95% CI = 2.13–31.27) in canned food users. The odds of having blood Cd levels in the upper tertile were 0.09 (95% CI = 0.01–0.96) for those being breastfed for 6–11 months compared to 0–5 months. Zn levels were lower (93.0 vs. 83.6 µg/dL, *p* = 0.008) in patients on a Phe-restricted diet. **Conclusions:** The present study did not find a relationship between heavy metal exposure and the dietary treatment status of patients with PKU. Our findings indicate that canned food consumption is a significant contributing factor to heavy metal exposure in PKU patients. Furthermore, our findings revealed a relationship between age, perception of economic level, breastfeeding, kitchen equipment, and water usage and the levels of certain heavy metals.

## 1. Introduction

Exposure to environmental pollutants usually results from direct or indirect human activities, and it can cause acute or chronic health problems. These pollutants include heavy metals as well as endocrine disruptors [1]. Exposure to environmental contaminants at early ages of life is of particular concern. Environmental exposure to toxic metals and chemicals is responsible for approximately 9 million deaths each year [2].

Heavy metals are a group of metals and metalloids that occur naturally [3]. Trace elements, on the other hand, are required by all living organisms. However, they can lead to serious health effects at both deficiency and high supplementation. While trace elements like manganese (Mn), copper (Cu), zinc (Zn), and selenium (Se) are needed to maintain normal growth, metabolism, and development, heavy metals such as lead (Pb), cadmium (Cd), arsenic (As), and mercury (Hg) are four of the top ten chemicals that threaten public health as reported by the World Health Organisation (WHO) [4,5]. As and Cd are categorized as “human carcinogen (Group 1)”; inorganic lead and lead compounds are classified as “probably carcinogenic to humans (Group 2A)”; methylmercury compounds are categorized as “possibly carcinogenic to humans (Group 2B)”; and metallic mercury and inorganic mercury compounds are “not classifiable to their carcinogenicity to humans (Group 3)” by the International Agency for Research on Cancer (IARC) [6,7,8,9]. Cd is classified as a “probable human carcinogen (Group B)”, As as a “human carcinogen (Group A)”, elemental Hg as “not classifiable as to human carcinogenicity (Group D)”, and methylmercury and mercuric chloride as possible human carcinogens (Group C) by the US Environmental Protection Agency (US EPA) [10,11,12,13]. In addition, heavy metal exposure has been reported to be associated with neurocognitive and behavioral disorders, respiratory problems, and cardiovascular diseases in children [14,15]. A number of studies conducted in various countries have documented the presence of detectable levels of heavy metals in soil and in a range of food products, including soil, fruit, vegetables, meat, milk, dairy products, and their canned counterparts [16,17,18,19]. Therefore, as oral intake is considered to be the main route of exposure to heavy metals as well as trace elements, the content and quality of the diet can affect both heavy metal intake and toxicity [20,21].

Phenylketonuria (PKU) is the most common amino acid metabolism disorder caused by a deficiency in the enzyme phenylalanine hydroxylase (PAH). The PAH enzyme converts phenylalanine (Phe) to tyrosine using tetrahydrobiopterin (BH4) as a cofactor. The PAH deficiency in untreated PKU patients leads to elevated Phe levels, which in turn causes intellectual impairment, eczematous rashes, seizures, motor function loss, and psychiatric problems [22,23]. While mild forms of PKU (blood Phe levels between 2 and 6 mg/dL) do not require treatment, patients with blood Phe levels of ≥6 mg/dL should be treated. The most well-established treatment, with a history of over six decades, is the Phe-restricted diet. In some patients, blood Phe levels can remain <6 mg/dL without the need for a Phe-restricted diet with sapropterin dihydrochloride therapy alone, an analog of BH4 [24,25]. Patients on a Phe-restricted diet may have very little or no protein-rich animal foods such as meat, milk, and eggs. Vegetables are an essential component of the daily diet for these patients; however, the unrestricted consumption of all vegetables is not feasible. The remainder of the daily protein requirement for the optimal growth and development of the patients on Phe-restricted (and therefore naturally protein-restricted) diets is provided by specially packaged medical Phe-free amino acid mixtures in powder or liquid form [23,26].

As the primary route of exposure to these heavy metals and trace elements is through the gastrointestinal system [14] and the diets of PKU patients vary according to treatment protocols, this study was designed with the aim of investigating whether the levels of plasma heavy metals and trace elements and their sources of exposure might differ between patients on different treatment regiments or not. The findings may provide insights into how the current PKU treatment protocols influence heavy metal and trace element exposure, potentially providing recommendations for improving these protocols in order to reduce exposure to high levels of heavy metals and trace elements.

## 2. Materials and Methods

### 2.1. Study Groups

This study was conducted concurrently in the same cohort as our investigation of plastic exposure in PKU [27]. Children aged 2–6 years, diagnosed with PKU through a newborn screening program and followed up at the pediatric metabolism center, were included in this descriptive analytic study between July 2022 and January 2024. The study groups were as follows:

1. Patients followed with hyperphenylalaninemia (HPA) (n = 38);
2. Patients with BH4-responsive PKU (n = 37);
3. Classical PKU or not responding to BH4 (n = 30).

In one of the patients in the HPA group, who did not respond to BH4 treatment, dietary intervention was initiated due to persistently elevated blood Phe levels above 6 mg/dL during follow-up. In the second group, three patients responded to BH4 treatment; however, their blood Phe levels remained above 6 mg/dL with the BH4 treatment alone, and dietary treatment was initiated. Consequently, 34 patients were on a Phe-restricted diet, while 71 patients were on an unrestricted diet.

Written informed consent was obtained from the participating children and their families before their enrollment into the study. The study’s inclusion criteria were as follows: diagnosis through a newborn screening program and regular follow-ups since then, the absence of chronic diseases other than PKU, and the absence of any other specialized diets in addition to the diet therapy provided within the scope of the PKU treatment.

A face-to-face file was administered to the parents of the participants to ascertain their socio-demographic characteristics, including age, birth order, total number of children, perception of economic status, family structure, and the education levels of both the mother and father. The survey was designed based on an evaluation of previous studies on environmental pollutants and covered various sources of exposure to heavy metals and trace elements, including parental smoking status, specific food consumption, water intake, and toys [28,29]. Information on the participants’ diseases (diagnosis, time of diagnosis, treatment modality, Phe levels) was also recorded through the hospital system.

Physical examinations and anthropometric measurements (height and weight) of all participants were taken and their body mass index z-scores (BAZ) were calculated.

### 2.2. Sample Collection

During the patients’ routine examinations, blood samples were collected in the morning in an EDTA tube. Plasma samples were obtained after centrifugating the samples at 3000 rpm for 10 min. Plasma samples were then stored at −20 °C until the analysis of Pb, As, Cd, Hg, Mn, Se, Cu, and Zn.

### 2.3. Chemicals

Nitric acid (65–69%, TraceMetal Grade), hydrogen peroxide (30–32%, TraceMetal Grade), and hydrochloric acid (35–37%, TraceMetal Grade) were obtained from Fisher Chemical (Hampton, NH, USA). Single element standard solutions (for all elements under study, each at 1000 µg/L) were obtained from Inorganic Ventures (Christiansburg, VA, USA). Gold ICP-MS Standard (1000 ppm Au in 3% HCl) was obtained from Fisher Chemical (Hampton, NH, USA).

### 2.4. Standard Preparation and Calibration

All the standard solutions for each metal (1000 µg/mL) were purchased from Inorganic Ventures (Christiansburg, VA, USA). Internal standards were also used. The individual calibration standards were prepared through serial dilution by diluting the five different groups of mixed working standards in the appropriate concentration ranges for linearity. An internal standard mixture was added to all samples at a concentration of 20 µg/L. Gold (200 µg/L) was added to all standards and rinse solutions to facilitate the washout of mercury. Deionized water (18.20 MΩ·cm) was obtained from a Thermo Scientific Barnstead MicroPure Water Purification System (Waltham, MA, USA).

### 2.5. Internal Standards

Pb: Lead (Pb) 208 (mass) Bismuth (Bi) 209 (mass)

Hg: Mercury (Hg) 202 (mass) Bismuth (Bi) 209 (mass)

As: Arsenic (As) 75 (mass) Germanium (Ge) 72 (mass)

Cd: Cadmium (Cd) 111 (mass) Rhodium (Rh) 103 (mass)

Mn: Manganese (Mn) 55 (mass) Scandium (Sc) 45 (mass)

Se: Selenium (Se) 76 (mass) Gadolinium (Gd) (152) (mass)

Cu: Copper (Cu) 65 (mass) Scandium (Sc) 45 (mass)

Zn: Zinc (Zn) 66 (mass) Scandium (Sc) 45 (mass)

### 2.6. Sample Preparation

The samples (100 µL) were taken in pre-cleaned, dry microwave digestion vessels. Gold was added to the samples at 200 µg/L as a final concentration in the sample solution to stabilize the mercury. Deionized water (2 mL), nitric acid (2 mL), hydrogen peroxide (1 mL), and hydrochloric acid (0.2 mL) were added to the samples, which were kept for 10 min to pre-digest in a fume hood. The microwave digestion vessels were closed, and the microwave digestion process was started with a set temperature program (ramp time: 40 min; hold time: 30 min; 190 °C, 1500 W). A CEM MARS™ 6 one-touch microwave digestion system (CEM Corporation, Matthews, NC, USA) was used to perform the microwave digestion. Later, the microwave digestion vessels were cooled by keeping the rotor at 25 °C for 15 min after the digestion process. The vessels were opened slowly and carefully put in a fume cupboard, so the pressurized acid fumes could evaporate. The digested sample solutions were quantitatively transferred to pre-cleaned volumetric flasks, with multiple rinses with deionized water. The internal standards were added from a stock solution containing 10 mg/L of each element (the final concentration was 30 µg/L), and the total volume was adjusted to a certain volume with deionized water. The prepared sample solutions were vortexed vigorously. A procedural blank was prepared without the sample matrix.

### 2.7. ICP-MS Analysis

The levels of Pb, As, Cd, Hg, Mn, Se, Cu, and Zn were measured by inductively coupled plasma-mass spectrometry (ICP-MS). The multi-elemental analysis of the plasma samples was performed using an iCAP RQ ICP-MS (Thermo Fischer Scientific, Waltham, MA, USA). To enable high-throughput analysis, a Teledyne autosampler (Teledyne CETAC Technologies, Omaha, NE, USA) was used. All analytes were measured using kinetic energy discrimination (KED) to assure the complete removal of polyatomic interference parameters, sample information, and applicable quality control tests. After the data acquisition was finished, the ICP-MS program was used to calculate the results for the unknown samples by using the data from the standards.

### 2.8. Linearity

The linearity is demonstrated using a six-point calibration curve. The R correlation coefficients for each metal were as follows: Pb: R^2^ = 0.999; As: R^2^ = 0.997; Cd: R^2^ = 0.998; Hg: R^2^ = 0.990; Mn: R^2^ = 0.991; Se: R^2^ = 0.994; Cu: R^2^ = 0.993; and Zn: R^2^ = 0.995.

### 2.9. Limit of Detections (LODs)

The limit of detections were as follows: Pb: 0.008 ppb; As: 0.07 ppb; Cd: 0.09 ppb; Hg: 0.11 ppb; Mn: 0.02 ppb; Se: 0.012 ppb; Cu: 0.025 ppb; and Zn: 0.013 ppb.

### 2.10. Recovery

When the recovery experiments were performed, the samples were spiked with analytes before the addition of an extraction solvent. The recovery values were as follows: Pb: 99%; As: 103%; Cd: 99%; Hg: 90%; Mn: 101%; Se: 102%; Cu: 97%; and Zn; 101%.

### 2.11. Statistical Analysis

The data were analyzed with IBM SPSS statistics software for Windows version 23.0 (Chicago, IL, USA). The distributions of the elements were examined with skewness, kurtosis, histograms, and the Kolmogorov–Smirnov test. In the intergroup comparisons, if there were a minimum of 10 cases (approximately 10% of the total number of cases) in each of the subgroups of independent variables, the differences between the groups were subjected to analysis. The heavy metals did not follow a normal distribution and were tested for differences using the Mann–Whitney U test for the binary variable subgroups and the Kruskal–Wallis one-way ANOVA for the variables with three subgroups. The trace elements followed a normal distribution and were analyzed using the independent sample *t*-test and ANOVA. The means with standard deviations (SDs), medians with interquartile ranges (IQRs), and percentages were provided where appropriate. For the heavy metals, the upper tertile levels were defined. The frequency of being in the upper tertile subgroup according to case characteristics was examined using the chi-square test. Independent variables with a *p*-value < 0.2 related to any heavy metal were included in the multiple logistic regression analysis. The likelihood of being in the upper tertile group for each heavy metal was examined (method: enter analysis) with the child–mother characteristics [Phe-restricted diet (ref: absence), child age (ref: ≥4 years), breastfeeding duration (ref: 0–5 mo), BAZ (ref: >1), perception of economic level (ref: income is more than expenses)] in Model 1. In Model 2, environmental risk factors [canned foods (ref: absence), canned beverages (ref: absence), buying new furniture in the last year (ref: absence), porcelain/ceramics use in food preparation and/or consumption (ref: absence), tap water used for cooking and/or drinking (ref: absence), spring water used for cooking and/or drinking (ref: absence), purified water used for cooking and/or drinking (ref: absence)] were added to Model 1. The odds ratios and 95% confidence intervals (CIs) were calculated. The relationship between the elements according to Phe restriction status was examined using Spearman correlation tests. In the comparisons, a *p*-value of <0.05 was considered statistically significant.

## 3. Results

A total of 105 patients who were diagnosed with the newborn screening program and continued their follow-ups with the pediatric metabolism outpatient clinic were included in the study. Of these patients, 34 (32.4%) were receiving Phe-restricted diet treatment, while 75 (67.6%) were on an unrestricted diet. The other baseline characteristics of the participants are described elsewhere [27]. The heavy metal exposure sources of the patients are given in Table 1.

The median levels of the heavy metals were 1.62 µg/dL for Pb, 0.42 µg/dL for As, 1.45 µg/L for Cd, and 3.68 µg/L for Hg. The median levels of the trace elements were 9.74 µg/L for Mn, 130.2 µg/L for Se, 105.2 µg/dL for Cu, and 89.4 µg/dL for Zn (Table 2).

### 3.1. Lead

The univariate analysis revealed no statistically significant differences in Pb levels among the participants according to their dietary regimens or other examined participant characteristics (Table S1). However, the binary analysis according to participant characteristics (Model 1) indicated an increase in the percentage of blood Pb levels in the upper tertile by 7.61 (95% CI = 1.73–33.52) in children who were breastfed for 12–23 months compared to those who were breastfed for 0–5 months. Upon adjusting the exposure sources with the baseline characteristics in the analysis (Model 2), it was determined that the increase was 6.20 times higher (95% CI = 1.19–32.34). The plasma levels of Pb were higher in those who consumed canned foods than in those who did not (*p* = 0.016). In the Model 2 multivariate analysis where the exposure sources were adjusted in addition to the baseline characteristics, it was observed that the percentage of individuals in the upper quartile of Pb levels increased by 3.47 times (95% CI = 1.07–11.29) in those who consumed canned foods, and the use of canned beverages was observed to have an odds ratio of 0.25 (95% CI = 0.07–0.89) for being in the upper tertile of Pb levels (Table 3).

### 3.2. Arsenic

There was no relationship between the plasma As levels and the baseline characteristics of the participants. The median plasma As level was observed to be higher in the participants who reported consuming canned foods (0.46 vs. 0.39 µg/dL, *p* = 0.004) and who used porcelain/ceramics for food preparation/eating (0.44 vs. 0.36 µg/dL, *p* = 0.044) compared to those who did not (Table S1). In Model 1, the percentage of participants under the age of four with As levels in the upper tertile was found to be 5.43 times (95% CI = 1.84–16.04) higher than that of participants over the age of four. It was observed that the odds of having As levels in the upper tertile were 0.22 (95% CI = 0.06–0.84) times higher for those in the middle income level compared to those in the high income level. Including the source of exposure in the analysis revealed that the percentage of As levels in the upper tertile increased by a factor of 7.26 (95% CI = 2.09–25.28) in individuals under four years of age. Notably, the economic level was not significantly effective in this context. It was observed that the percentile of As levels in the upper tertile of canned food users increased by 8.17 times (95% CI = 2.13–31.27) in Model 2 (Table 3).

### 3.3. Cadmium

The median plasma Cd levels were significantly lower in the group that used purified water (1.53 vs. 1.37 µg/L, *p* = 0.02) (Table S1). Although no correlation was observed between the plasma Cd levels and the participants’ baseline characteristics in the primary analysis, a correlation was found between breastfeeding duration and economic status in the multivariate analysis. In Model 1, the odds of having blood Cd levels in the upper tertile were 0.05 times (95% CI = 0.01–0.49) for those being breastfed for 6–11 months compared to 0–5 months. This effect persisted in Model 2 (AOR = 0.09, 95% CI = 0.01–0.96). The results indicated that the participants with a mid-range economic status were more likely to have plasma Cd levels in the upper tertile percentile, with an odds ratio of 0.26 (95% CI = 0.07–0.99) in Model 1 and 0.22 (95% CI = 0.05–0.98) in Model 2, compared to those with a high economic status (Table 3).

### 3.4. Mercury

The participants living in homes where new furniture had been purchased in the last year had higher median plasma Hg levels (4.02 vs. 3.65 µg/L, *p* = 0.017; Table S1). These increases were not statistically significant in Model 2. The odds of having Hg levels in the upper tertile in those with a body mass index (BMI) for age z-score standard deviation score (BAZ SDS) between −1 and 1 were 3.30 times higher (95% CI = 1.18–9.23) compared to those with a BAZ SDS ≥ 1. This increase was observed as 3.10 times higher (95% CI = 1.00–9.62) in Model 2 (Table 3).

### 3.5. Trace Elements

The reference ranges used in our laboratory for the trace elements were 4–15 µg/L for Mn, 70–150 µg/L for Se, 70–160 µg/dL for Cu, and 63–118 µg/dL for Zn. The percentages of participants within normal blood ranges for Mn, Se, Cu, and Zn were 100%, 91%, 98%, and 93%, respectively.

No association was identified between the plasma Mn levels and any participant characteristics. The mean Cu levels were higher in those <4 years of age (108.4 vs. 101.5 µg/dL, *p* = 0.033) and in those who consumed frozen foods (109.2 vs. 102.6 µg/dL, *p* = 0.047), and were lower in those who used porcelain/ceramics to prepare and/or eat food (103.8 vs. 113.4 µg/dL, *p* = 0.029). The mean Zn levels were lower (93.0 vs. 83.6 µg/dL, *p* = 0.008) in those on a Phe-restricted diet compared to those on an unrestricted diet. The mean plasma Cu levels were higher in the participants who reported the use of frozen foods compared to those who did not (109.2 vs. 102.6 µg/dL, *p* = 0.047), and lower in participants who reported the use of porcelain/ceramic equipment in food preparation and eating compared to those who did not (103.8 vs. 113.4 µg/dL, *p* = 0.029). The mean plasma Zn levels were higher in those who used bottled water for food/drinking (94.4 vs. 87.3, *p* = 0.038) (Table 4).

### 3.6. Correlation between Toxic and Trace Elements According to Dietary Status

In the patients receiving the Phe-restricted diet treatment, a moderately strong positive correlation (r_s_ = 0.43) was observed between Pb and As, and a weak positive correlation was noted between Pb and Mn (r_s_ = 0.39) and between Cd and Hg (r_s_ = 0.39). In the unrestricted diet group, a moderately strong relationship (rs = 0.43) was observed between Pb and As, while no correlation was identified between P and Mn. In this group, a moderately strong positive correlation was also identified between Zn and As (r_s_ = 0.45), between Mn and Cd (r_s_ = 0.28), and between Zn and Pb (r_s_ = 0.31). Furthermore, a moderately strong negative correlation was observed between Se and Mn (r_s_ = −0.45), and a weak negative correlation was observed between Se and As (r_s_ = −0.28), Cd (r_s_ = −0.24), and Zn (r_s_ = −0.33) (Table 5).

## 4. Discussion

This study is the first in the literature to evaluate the exposure of patients with PKU to multiple heavy metals and the correlation of the heavy metals with trace elements. In a separate study conducted on the same cohort, we observed that the exposure of PKU patients to certain plasticizers varied according to their dietary status. However, in the present study, we found that the exposure of PKU patients to heavy metals did not vary according to their nutritional status or treatment regimen [27]. Furthermore, the patients who were on BH4 therapy or on a Phe-restricted diet did not face an elevated risk of exposure to toxic elements due to their treatment compared to those without treatment.

The Centers for Disease Control and Prevention (CDC) has set the upper limit (UL) for blood Pb levels in children 1 to 5 years of age at 3.5 µg/dL by 2021 [30]. The maximum level in our study was 3.02, below the specified UL. The blood Pb levels observed in this study were comparable to those reported in healthy children in previous studies conducted in Türkiye [31,32]. The median blood As level of the patients (0.42 µg/dL) was below the UL of 1 µg/dL, given by the Agency for Toxic Substances and Disease Registry (ATSDR) [33]. A comparison could not be made due to the absence of studies in the existing literature that assess blood As levels in Türkiye within the specified age group. The blood As levels were reported as 0.10 µg/L in children with an average age of 5 µg/L in China and 1.36 µg/L in a study evaluating 60 Peruvian children aged 3–24 months [34,35]. According to National Health and Nutrition Examination Survey (NHANES) data, although the 95th percentile of blood Cd levels in children aged 1–5 years varies by 0.2–0.4 µg/L depending on age, ≤0.5 µg/L is generally accepted as a safe level for the blood Cd level in children [36]. In our study, we found that the median blood Cd level of the patients was 1.44 µg/L, which is above this value. Bayhan et al. (2017) reported a mean blood Cd level of 0.58 µg/L in healthy individuals with a mean age of 16.8 years in Türkiye [37]. In another study conducted by Yalçın et al. (2022), the mean blood Cd level of children with a mean age of 8.2 years was 0.18 µg/L, and the mean blood Hg level was 0.16 µg/L [38]. In our study, the median blood Hg value of the participants was 3.68 μg/L. According to the 2005–2006 NHANES data, the 95% p for the blood Hg levels of children aged 1–5 years was 1.43 μg/dL (14.3 μg/L), and this value increases with age [39]. The levels of heavy metals, particularly those of As and Cd, were found to be higher than those reported in other studies conducted in Türkiye. Nevertheless, the levels of these metals did not vary in accordance with the patients’ diagnoses, treatments, and mean Phe levels. This indicates that the elevated levels of As and Cd observed in this study are attributable to regional and individual exposure variations rather than to the underlying disease.

### 4.1. Lead

A comparative analysis revealed that the levels of heavy metals were higher in the individuals who consumed canned foods. In a series of examinations conducted by the Food and Drug Administration (FDA) on 3276 food samples (including canned foods), the presence of Pb, As, Cd, and Hg was identified in 86%, 57%, 39%, and 93% of the samples, respectively [40]. In the evaluation of the dietary exposure to heavy metals in Japan, As has been detected in all samples of twelve out of fourteen food groups studied, Cd in ten, and Pb in eight [41]. In a review of the literature, Brhane and colleagues (2014) suggested that the presence of Pb in canned foods was above the tolerable intake level [42]. The presence of heavy metals in canned foods in Jordan has been identified, with concentrations exceeding the limits set by various health organizations [17]. Several studies exist in the literature that indicate the presence of heavy metals in protein-rich foods, including meat, meat products, fish, milk, and dairy products, as well as canned vegetables [43,44,45]. In line with the existing literature, our study found that the individuals who consumed canned foods had higher levels of heavy metal exposure. Given that this exposure did not vary according to dietary status, it can be postulated that the heavy metal exposure could have occurred through both canned meat and meat products and canned vegetables. Nevertheless, it is not feasible to reach a definitive conclusion due to the design of the study.

The results of our study indicated that the blood Pb levels of the infants who were breastfed for a total period of between 12 and 24 months were higher than those of the infants who were breastfed for a shorter period. More than 90% of the Pb in the human body is stored in the bones [46]. Lead (Pb) competes with calcium (Ca) in the formation of apatite crystals in the bone tissue. As the Pb replaces the Ca, the resulting bone tissue becomes osteoporotic [47,48,49]. Bone turnover increases during breastfeeding due to the increased calcium demand and other hormonal changes [50]. In addition to the increased bone turnover that occurs as a result of the physiological and hormonal changes during the lactation period, the turnover of the bone tissue with reduced mineral content, caused by the mother’s exposure to Pb, increases even further in order to meet the increased Ca need. Consequently, more Pb is released into the mother’s blood and subsequently into the breast milk. Actually, this release is greater during lactation than pregnancy [51,52]. Nevertheless, one study indicated that the Pb levels in breast milk accounted for only 12% of the observed variation in infant blood Pb levels [53]. In a separate study that examined the correlation between cord blood, breast milk, and infant hair Pb levels, no statistically significant relationship was identified [29]. In addition, the difference in the Pb levels was only seen in those who breastfed for up to 12–24 months. In the specified age range, the infants were consuming complementary foods in addition to the breast milk. This could have been attributed to the Pb exposure resulting from personal food preferences rather than the dietary treatment. However, the complementary food intake patterns were comparable among the infants who were breastfed for 12 months or longer in Türkiye [54]. The available evidence indicates that the elevated blood Pb levels observed in children who have been breastfed for longer periods are not a direct consequence of the breast milk itself, but rather the result of the Pb exposure of their mothers. Furthermore, the CDC advises that infant blood Pb levels should be monitored in cases where the maternal blood Pb levels are between 5 and 39 μg/dL, and that breastfeeding should be continued in instances where the infant blood Pb levels are <5 μg/dL. The blood Pb levels of our patients during the breastfeeding period are unknown. However, if we accept the measured level as a reflection of the early blood Pb levels, it can be posited that the blood Pb levels of all our patients were nearly half of 5 μg/dL, and thus, no changes regarding breastfeeding would probably be needed.

The plasma Pb levels of those who consumed canned beverages were observed to be lower than in those who did not consume them. This finding was not anticipated. Two separate studies conducted by Yüksel et al. (2023) and Bingöl et al. (2010) in Türkiye showed that the Pb level in canned non-alcoholic beverages was below the maximum permissible level [55,56]. Furthermore, studies conducted in various countries have indicated that the levels of Pb present in canned beverages are relatively low [57,58,59]. However, other studies documented elevated levels of Pb in canned beverages [60,61].

We found a positive correlation between spring water use and plasma Pb levels. The findings of studies conducted in Türkiye, which revealed that natural water Pb levels exceeded the permissible limits for drinking water, are consistent with our own results [62,63,64].

### 4.2. Arsenic

In the multivariate analysis (Model 2), a correlation was identified between plasma As levels and age, as well as canned food consumption. Children are exposed to more environmental pollutants, including heavy metals, for reasons such as putting toys, etc., in their mouths as an exploratory behavior, having a greater surface area/volume ratio than adults, having living spaces closer to the ground, and their gastrointestinal absorption frequently increasing due to their rapid growth rate, and these risks increase with age [65,66]. The low plasma As levels in patients < 4 years of age may be due to these reasons.

### 4.3. Cadmium

The sources of exposure found to be associated with plasma Cd levels in the primary analyses were not significant in further analyses. In the multivariate analysis, a significant association was found only with the baseline characteristics, economic status, and duration of breastfeeding. In studies conducted in Türkiye, Gürbay et al. (2012) reported that Cd was detected in only one of the 64 breast milk samples analyzed, while Örün et al. (2011) reported that the median Cd level in the breast milk they analyzed was 0.67 μg/L [67,68]. Cd levels in breast milk were reviewed by Rebelo et al. (2016) in 28 studies, and the Cd levels were found to be quite low [69]. In one study conducted by Bassil et al. (2018) that evaluated the presence of heavy metals in breast milk, it was reported that the lowest concentration of the toxic elements detected was that of Cd [70]. It can be concluded that breast milk does not present a risk in terms of Cd exposure. Nevertheless, infants who are breastfed for shorter periods will be exposed at an earlier age to foods that may act as potential sources of Cd exposure or to infant formulas prepared with contaminated water [71]. These findings collectively indicate that extended breastfeeding may confer protection against Cd exposure. Nevertheless, this effect was not observed in participants who breastfed for a duration exceeding 12 months. Between 6 and 12 months, infants can get about half of their daily energy needs from breast milk. After 12 months, only one-third of their daily energy needs come from breast milk, and this rate decreases over time. Complementary feeding is started at 6 months, and the amount of foods other than breast milk taken daily is gradually increased [72]. Probably for the reasons discussed above, infants become more exposed to Cd sources as they grow older, and the advantageous effect of breastfeeding on the Cd exposure disappears.

### 4.4. Mercury

A negative correlation was identified between the blood Hg levels and BAZ. While some studies have indicated a positive correlation between blood Hg levels and obesity [73,74,75], others have demonstrated that Hg exposure may actually lead to a reduction in adipose tissue [76,77,78]. Furthermore, the utilization of porcelain/ceramic equipment in the preparation and/or consumption of food items was observed to have a positive correlation with the blood Hg levels. In the absence of data on this relationship in the existing literature, it is not possible to offer an informed commentary.

### 4.5. Trace Elements

The plasma Zn levels were lower in those receiving a Phe-restricted diet (93.0 vs. 83.6 µg/dL, *p* = 0.008). Animal foods are rich in Zn, and Zn deficiency is a common condition in people who have a limited intake of animal foods [79]. Patients on Phe-restricted diet therapy receive Phe-free amino acid mixtures to meet the protein needs for optimal growth and development. Some micronutrients, whose main source is animal foods, are also provided by the amino acid mixtures. However, these patients are more susceptible to micronutrient deficiencies for reasons such as not using sufficient amino acid mixture, not adhering to the diet but also not consuming protein-containing foods, or having a diet limited to only natural protein in mild forms of the disease [80]. Zn deficiency has been reported for many years in PKU patients on dietary therapy [81,82]. It has been reported that some patients who take adequate amounts of Zn in amino acid mixtures still develop Zn deficiency [83]. This is likely attributable to the reduced bioavailability of Zn resulting from its intake in the amino acid mixtures as opposed to natural proteins [80].

The plasma Se levels were higher in those on a Phe-restricted diet than in those on an unrestricted diet. Se is an element that has very important functions in the oxidative and anti-oxidative systems in the human body [84]. Moreover, Se plays a role in a number of biological processes, including the immune system, thyroid hormone production, and reproductive system [85,86]. The primary source of Se is dietary intake, with the concentration of this mineral in foods largely dependent on the Se content of the soil in which they are cultivated. While the Se content of vegetables is typically lower than that of animal foods, certain plant-based foods, including onions, potatoes, peppers, peas, and garlic, can contain notable levels of Se [87,88]. Despite our initial expectation that the plasma Se levels would be higher in the unrestricted diet group due to their higher consumption of animal products, the results of our study indicated a discrepancy between this expectation and the observed outcome. One potential explanation for this discrepancy is that the patients consumed a greater quantity of vegetables rich in Se. An additional explanation is that the amino acid mixtures used by the patients on a Phe-restricted diet contain sufficient amounts of Se, which leads to the conclusion that patients using the amino acid mixtures are receiving sufficient amounts of Se. Despite this fact, it is possible that the Se levels of the patients on a free diet may have been lower, depending on their food preferences. However, since the patients’ detailed nutritional content was not questioned, we cannot provide any data to support this idea.

### 4.6. Correlation between Heavy Metals and Trace Elements According to Dietary Status

In the Phe-restricted diet group, correlations were found between Pb and As, Pb and Mn, and Cd and Hg. In the unrestricted diet group, a similar correlation was found between Pb and As. In other words, diet did not make a difference in the relationship between Pb and As. It is of particular interest to highlight the negative relationship between Se and heavy metals observed in the unrestricted diet group. In the unrestricted diet group, a negative correlation was found with As and Cd, meaning that high levels of these toxic elements are associated with low levels of Se. As the As and Cd exposure increases in these patients, they will face additional potential health risks due to the low Se levels in addition to the potential harms of these toxic elements.

## 5. Conclusions

This study is the first in the literature to examine the exposure of patients with PKU to heavy metals and the status of this exposure according to diet. The present study did not find a relationship between heavy metal exposure and the dietary treatment status of patients with PKU. Our findings indicate that canned food consumption is a significant contributing factor to the heavy metal exposure in PKU patients. Furthermore, our findings revealed a relationship between age, perception of economic level, breastfeeding, kitchen equipment, and water usage and the levels of certain toxic elements. Further research is required to gain a more comprehensive understanding of the environmental pollutant exposure of patients with PKU and to enable the implementation of effective strategies to minimize their exposure.

## Figures and Tables

**Table 1:** The possible sources of heavy metal exposure for all patients.

	Number, (%)
*Parent’s smoking status*	
At least one of	65 (61.9)
None of	40 (38.1)
*Baby shampoo*	94 (89.5)
*Frozen foods*	44 (41.9)
*Canned foods*	57 (54.3)
*Packaged foods*	101 (96.2)
*Canned beverages*	40 (38.1)
*Cartridge printer*	9 (8.6)
*Buying new furniture in the last year*	24 (22.9)
*Using glass when preparing and/or eating food*	47 (44.8)
*Using porcelain/ceramics when preparing and/or eating food*	88 (83.8)
*Water used for cooking and/or drinking*	
Tap water	64 (61.0)
Spring water	14 (13.3)
Bottled water	39 (37.1)
Purified water	30 (28.6)
*Toys*	
Plastic	103 (98.1)
Painted wood	44 (41.9)
Unpainted wood	59 (56.2)
Plush	88 (83.8)

**Table 2:** Plasma heavy metal and trace element distributions for all patients.

		Percentiles						
	GM	10	25	33	50	67	75	90
*Heavy metals*								
Pb, µg/dL	1.50	0.88	1.08	1.18	1.62	2.03	2.11	2.38
As, µg/dL	0.42	0.27	0.33	0.37	0.42	0.48	0.51	0.57
Cd, µg/L	1.44	1.06	1.27	1.35	1.45	1.62	1.66	1.87
Hg, µg/L	3.86	3.23	3.48	3.56	3.68	4.04	4.27	4.87
*Trace elements*								
Mn, µg/L	9.78	8.28	8.86	9.17	9.74	10.48	10.67	11.64
Se, µg/L	129.5	110.6	119.9	123.4	130.2	137.0	140.6	150.3
Cu, µg/dL	104.0	83.6	93.8	97.8	105.2	112.7	116.7	127.4
Zn, µg/dL	88.4	67.3	76.9	79.3	89.4	95.2	103.7	110.3

As: arsenic, Cd: cadmium, Cu: copper, Hg: mercury, Mn: manganese, Pb: lead, Se: selenium, Zn: zinc. GM: geometric mean.

**Table 3:** Association between child characteristics and sources of exposure and upper tertiles of heavy metals by multivariate regression analysis.

	Dependent Variable							
	Pb > 2 µg/dL		As > 0.48 µg/dL		Cd > 1.6 µg/L		Hg > 4 µg/L	
	AOR (95% CI)		AOR (95% CI)		AOR (95% CI)		AOR (95% CI)	
	Model 1 ^&^	Model 2 ^#^	Model 1 ^&^	Model 2 ^#^	Model 1 ^&^	Model 2 ^#^	Model 1 ^&^	Model 2 ^#^
Child Characteristics								
*Diet*								
Unrestricted diet	1.00	1.00	1.00	1.00	1.00	1.00	1.00	1.00
Phe-restricted diet	0.48 (0.16–1.43)	0.63 (0.18–2.23)	0.38 (0.13–1.19)	0.50 (0.14–1.79)	1.06 (0.36–3.13)	0.90 (0.26–3.12)	1.56 (0.60–4.08)	2.09 (0.68–6.38)
*Age*								
≥4 years	1.00	1.00	1.00	1.00	1.00	1.00	1.00	1.00
<4 years	2.48 (0.93–6.59)	2.78 (0.93–8.34)	5.43 (1.84–16.04)	7.26 (2.09–25.28)	2.10 (0.81–5.47)	2.26 (0.76–6.73)	0.80 (0.32–2.02)	0.82 (0.29–2.28)
*BAZ*								
≥1 SDS	1.00	1.00	1.00	1.00	1.00	1.00	1.00	1.00
≤−1 SDS	0.61 (0.11–3.31)	0.43 (0.05–3.68)	1.24 (0.24–6.56)	0.69 (0.08–5.84)	0.24 (0.04–1.45)	0.19 (0.03–1.42)	0.87 (0.14–5.32)	0.75 (0.11–5.12)
−1< <1 SDS	1.36 (0.48–3.86)	1.85 (0.57–6.05)	0.90 (0.30–2.68)	0.67 (0.20–2.27)	0.49 (0.18–1.35)	0.45 (0.14–1.51)	3.30 (1.18–9.23)	3.10 (1.00–9.62)
*Perception of economic level*								
Income is more than expenses	1.00	1.00	1.00	1.00	1.00	1.00	1.00	1.00
Income is less than expenses	1.29 (0.34–4.88)	1.81 (0.39–8.41)	0.65 (0.18–2.34)	1.07 (0.24–4.77)	0.47 (0.13–1.72)	0.72 (0.16–3.25)	1.03 (0.30–3.60)	1.07 (0.27–4.21)
Income is equal to expenses	0.57 (0.16–2.05)	0.72 (0.18–2.89)	0.22 (0.06–0.84)	0.34 (0.08–1.43)	0.26 (0.07–0.99)	0.22 (0.05–0.98)	1.55 (0.46–5.25)	1.69 (0.45–6.32)
*Total breastfeeding time*								
0–5 months	1.00	1.00	1.00	1.00	1.00	1.00	1.00	1.00
6–11 months	1.70 (0.34–8.41)	1.74 (0.29–10.38)	0.28 (0.06–1.39)	0.27 (0.04–1.71)	0.05 (0.01–0.49)	0.09 (0.01–0.96)	1.04 (0.26–4.12)	1.71 (0.35–8.38)
12–23 months	7.61 (1.73–33.52)	6.20 (1.19–32.34)	1.32 (0.33–5.36)	1.53 (0.28–8.37)	1.55 (0.43–5.58)	3.41 (0.70–16.60)	0.60 (0.17–2.15)	0.49 (0.12–2.06)
24 months or more	2.64 (0.54–11.23)	1.17 (0.22–6.36)	0.38 (0.09–1.65)	0.26 (0.05–1.51)	1.01 (0.26–3.98)	2.08 (0.39–11.26)	0.37 (0.09–1.53)	0.39 (0.08–1.93)
*Canned foods*								
No		1.00		1.00		1.00		1.00
Yes		3.47 (1.07–11.29)		8.17 (2.13–31.27)		2.79 (0.87–8.96)		1.84 (0.66–5.12)
*Canned beverages*								
No		1.00		1.00		1.00		1.00
Yes		0.25 (0.07–0.89)		0.68 (0.20–2.34)		2.45 (0.73–8.20)		0.93 (0.33–2.65)
*Buying new furniture in the last year*								
No		1.00		1.00		1.00		1.00
Yes		1.09 (0.28–4.20)		1.60 (0.43–5.96)		3.12 (0.88–11.01)		2.36 (0.78–7.13)
*Porcelain/ceramics equipment for use in food preparation and/or consumption*								
No		1.00		1.00		1.00		1.00
Yes		2.41 (0.48–11.98)		1.89 (0.37–9.66)		0.63 (0.12–3.41)		4.80 (1.02–22.67)
*Tap water used for cooking and/or drinking*								
No		1.00		1.00		1.00		1.00
Yes		1.74 (0.40–7.59)		0.86 (0.20–3.74)		0.60 (0.14–2.56)		1.10 (0.32–3.80)
*Spring water used for cooking and/or drinking*								
No		1.00		1.00		1.00		1.00
Yes		7.29 (1.21–44.03)		1.55 (0.25–9.49)		1.97 (0.37–10.48)		2.37 (0.49–11.50)
*Purified water used for cooking and/or drinking*								
No		1.00		1.00		1.00		1.00
Yes		3.77 (0.74–19.22)		3.09 (0.60–16.02)		0.23 (0.04–1.23)		1.42 (0.35–5.68)
*Painted wood toys*								
No		1.00		1.00		1.00		1.00
Yes		1.62 (0.55–4.78)		1.43 (0.46–4.51)		1.89 (0.63–5.68)		0.55 (0.20–1.51)

^&^ Adjusted for age, BMI for age z-score (BAZ), diet status, perception of economic level, and total breastfeeding time; ^#^ adjusted for Model 1 and exposure sources. AOR: adjusted odds ratio, As: arsenic, Cd: cadmium, Hg: mercury, Pb: lead.

**Table 4:** Relationship between participant characteristics and trace elements in all patients.

	Mn ^¥^	*p*	Se ^¥^	*p*	Cu ^¥^	*p*	Zn ^¥^	*p*
*Phe-restricted diet*								
Yes (n = 34)	9.69 ± 1.25	0.345	133.8 ± 11.6	0.067	107.0 ± 17.4	0.490	83.6 ± 15.5	0.008
No (n = 71)	9.97 ± 1.43		128.7 ± 16.2		104.6 ± 16.3		93.0 ± 17.0	
*Sex*								
Girl (n = 56)	9.69 ± 1.35	0.136	132.0 ± 15.0	0.221	103.37 ± 16.38	0.191	90.8 ± 17.6	0.559
Boy (n = 49)	10.09 ± 1.38		128.4 ± 15.0		107.64 ± 16.85		88.9 ± 16.4	
*Age*								
<4 years (n = 59)	10.02 ± 1.32	0.215	128.4 ± 15.9	0.139	108.4 ± 17.9	0.033	92.7 ± 16.9	0.063
≥4 years (n = 46)	9.69 ± 1.44		132.8 ± 13.7		101.5 ± 14.2		86.4 ± 16.7	
*BAZ*								
≤−1 (n = 11)	10.23 ± 1.62	0.662	127.0 ± 15.9	0.548	104.2 ± 16.3	0.713	98.4 ± 17.3	0.144
−1 < < 1 (n = 64)	9.82 ± 1.26		129.9 ± 12.6		104.6 ± 17.6		90.0 ± 17.3	
≥1 SDS (n = 30)	9.87 ± 1.54		132.5 ± 19.2		107.5 ± 14.9		86.7 ± 15.7	
*Diagnosis*								
HPA (n = 38)	10.11 ± 1.28	0.423	128.8 ± 18.0	0.618	103.5 ± 14.4	0.662	92.9 ± 18.3	0.113
PKU (n = 30)	9.72 ± 1.19		132.4 ± 11.5		107.2 ± 17.0		84.6 ± 16.0	
BH4 responsive (n = 37)	9.76 ± 1.60		130.2 ± 14.4		105.8 ± 18.7		91.3 ± 15.9	
*Birth order*								
First child (n = 50)	9.97 ± 1.18	0.506	130.5 ± 15.5	0.942	102.8 ± 17.3	0.137	91.4 ± 16.1	0.391
1 ≥ 2. child (n = 55)	9.79 ± 1.54		130.2 ± 14.8		107.7 ± 15.9		88.6 ± 17.8	
*Total number of children*								
1 (n = 43)	9.88 ± 1.17	0.390	131.9 ± 15.8	0.258	104.4 ± 17.5	0.471	91.7 ± 16.1	0.668
2 (n = 38)	9.68 ± 1.48		127.1 ± 14.1		104.1 ± 15.4		88.7 ± 18.1	
3 and more (n = 24)	10.18 ± 1.54		132.6 ± 14.9		109.0 ± 17.3		88.7 ± 17.2	
*Family structure*								
Nuclear (n = 89)	9.92 ± 1.40	0.479	130.1 ± 14.6	0.646	105.2 ± 16.9	0.771	89.3 ± 16.8	0.333
Extended (n = 16)	9.65 ± 1.23		131.9 ± 17.9		106.5 ± 15.9		93.7 ± 18.2	
*Perception of economic level*								
Income is less than expenses (n = 33)	9.52 ± 1.50	0.187	130.3 ± 14.3	0.144	103.7 ± 16.4	0.540	86.1 ± 18.8	0.265
Income is equal to expenses (n = 53)	10.0 ± 1.44		132.5 ± 16.6		105.1 ± 16.8		91.1 ± 16.0	
Income is more than expenses (n = 19)	10.16 ± 0.73		124.6 ± 9.8		109.0 ± 17.1		93.3 ± 16.2	
*Total breastfeeding time, month*								
0–5 (n = 24)	9.52 ± 1.03	0.102	130.7 ± 14.0	0.362	107.8 ± 15.2	0.370	87.8 ± 19.3	0.661
6–11 (n = 22)	9.52 ± 1.35		132.8 ± 14.0		108.6 ± 18.1		87.3 ± 13.3	
12–23 (n = 29)	10.04 ± 1.62		132.4 ± 17.8		105.1 ± 18.3		92.3 ± 16.7	
24 and more (n = 39)	10.28 ± 1.31		126.3 ± 13.5		101.3 ± 15.0		91.3 ± 18.1	
*Maternal education level*								
≤8 years (n = 64)	9.84 ± 1.46	0.730	131.8 ± 15.3	0.242	105.3 ± 17.6	0.988	90.6 ± 17.1	0.637
>8 years (n = 41)	9.93 ± 1.26		128.2 ± 14.6		105.4 ± 15.3		89.0 ± 17.0	
*Paternal education level*								
≤8 years (n = 64)	9.76 ± 1.45	0.285	131.8 ± 15.0	0.226	104.9 ± 16.9	0.751	89.5 ± 17.5	0.745
>8 years (n = 41)	10.06 ± 1.25		128.1 ± 15.0		106.0 ± 16.5		90.6 ± 16.4	
*Parent’s smoking status*								
One or both of (n = 65)	9.82 ± 1.45	0.587	130.6 ± 14.3	0.829	107.1 ± 16.4	0.175	89.5 ± 16.9	0.655
None of (n = 40)	9.97 ± 1.26		129.9 ± 16.3		102.5 ± 16.9		90.9 ± 17.4	
*Frozen foods*								
Yes (n = 44)	9.97 ± 1.49	0.565	129.8 ± 16.2	0.647	102.6 ± 16.0	0.047	86.5 ± 15.1	0.079
No (n = 61)	9.81 ± 1.30		131.1 ± 13.4		109.2 ± 17.0		92.4 ± 18.0	
*Canned foods*								
Yes (n = 57)	10.07 ± 1.31	0.109	132.1 ± 14.6	0.278	104.6 ± 16.4	0.678	92.7 ± 15.5	0.074
No (n = 48)	9.64 ± 1.43		128.9 ± 15.4		106.0 ± 17.0		86.7 ± 18.3	
*Canned beverages*								
Yes (n = 40)	9.84 ± 1.39	0.847	130.6 ± 15.2	0.799	103.3 ± 16.6	0.109	88.6 ± 17.8	0.532
No (n = 65)	9.90 ± 1.38		129.86 ± 15.0		108.7 ± 16.5		90.8 ± 16.6	
*Buying new furniture in the last year*								
Yes (n = 24)	10.19 ± 1.42	0.212	130.8 ± 14.7	0.553	105.2 ± 16.0	0.876	89.9 ± 16.8	0.983
No (n = 81)	9.79 ± 1.36		128.7 ± 16.4		105.8 ± 19.1		90.0 ± 17.2	
*Equipment used for food preparation and/or consumption*								
Glass								
Yes (n = 47)	10.02 ± 1.40	0.347	130.0 ± 14.2	0.820	104.1 ± 17.3	0.372	91.4 ± 17.1	0.419
No (n = 58)	9.76 ± 1.36		130.7 ± 16.2		107.0 ± 15.9		88.7 ± 16.9	
Porcelain								
Yes (n = 88)	9.81 ± 1.27	0.266	127.9 ± 13.9	0.470	113.4 ± 17.1	0.029	90.1 ± 17.6	0.822
No (n = 17)	10.22 ± 1.83		130.8 ± 15.3		103.8 ± 16.2		89.1 ± 14.0	
*Water used for cooking and/or drinking*								
Tap water								
Yes (n = 64)	10.03 ± 1.19	0.187	131.5 ± 13.1	0.550	105.5 ± 17.2	0.932	91.5 ± 18.4	0.249
No (n = 41)	9.64 ± 1.62		129.6 ± 16.2		105.3 ± 16.4		87.5 ± 14.3	
Spring water								
Yes (n = 14)	10.38 ± 1.63	0.139	129.6 ± 14.3	0.174	104.9 ± 16.4	0.479	94.3 ± 15.4	0.305
No (n = 91)	9.80 ± 1.33		135.4 ± 19.2		108.3 ± 18.4		89.3 ± 17.2	
Bottled water								
Yes (n = 39)	9.75 ± 1.22	0.455	131.3 ± 15.8	0.406	106.4 ± 16.1	0.397	94.4 ± 18.1	0.038
No (n = 66)	9.95 ± 1.46		128.7 ± 13.7		103.6 ± 17.6		87.3 ± 15.8	
Purified water								
Yes (n = 30)	9.60 ± 1.64	0.190	131.5 ± 15.5	0.201	104.6 ± 17.4	0.467	87.3 ± 17.9	0.310
No (n = 75)	9.99 ± 1.25		127.4 ± 13.7		107.2 ± 14.7		91.0 ± 16.6	
*Toys*								
Painted wood								
Yes (n = 44)	10.18 ± 1.31	0.054	131.5 ± 14.1	0.365	104.6 ± 16.3	0.562	89.0 ± 18.7	0.641
No (n = 61)	9.66 ± 1.39		128.8 ± 16.2		106.5 ± 17.2		90.6 ± 15.8	
Unpainted wood								
Yes (n = 59)	9.80 ± 1.35	0.501	130.6 ± 16.0	0.860	106.7 ± 18.7	0.461	91.1 ± 17.9	0.414
No (n = 46)	9.98 ± 1.41		130.1 ± 14.3		104.3 ± 14.9		88.4 ± 15.9	
Plush								
Yes (n = 88)	9.95 ± 1.38	0.235	134.4 ± 15.1	0.230	111.5 ± 15.3	0.098	90.4 ± 17.3	0.502
No (n = 17)	9.51 ± 1.34		129.6 ± 15.0		104.2 ± 16.7		87.4 ± 15.7	

Given as ^¥^ mean (SD). BAZ: BMI for age z-score, BH4: tetrahydrobiopterin, Cu: copper, HPA: hyperphenylalaninemia (blood phenylalanine levels within 2–6 mg/dL without any treatment), Mn: manganese, PKU: phenylketonuria, Phe: phenylalanine, Se: selenium, Zn: zinc. *p*-value < 0.05 was considered to be statistically significant.

**Table 5:** Correlation between toxic and trace elements according to dietary status.

			Phe-Restricted Diet							
			Pb	As	Cd	Hg	Mn	Se	Cu	Zn
Unrestricted diet	Pb	r_s_		0.43	0.21	0.04	0.39	−0.02	−0.03	−0.14
		p		0.010	0.227	0.837	0.023	0.893	0.861	0.424
	As	r_s_	0.43		−0.07	−0.10	0.23	0.11	−0.20	0.07
		p	0.010		0.686	0.565	0.186	0.530	0.268	0.702
	Cd	r_s_	−0.01	0.18		0.39	0.24	−0.13	0.05	0.18
		p	0.938	0.131		0.021	0.172	0.473	0.771	0.308
	Hg	r_s_	0.14	−0.02	0.14		0.20	0.07	−0.08	0.27
		p	0.230	0.883	0.230		0.261	0.691	0.654	0.126
	Mn	r_s_	0.14	0.22	0.28	−0.02		−0.18	0.27	0.06
		p	0.256	0.066	0.018	0.900		0.297	0.122	0.745
	Se	r_s_	−0.04	−0.28	−0.24	0.06	−0.45		−0.08	0.16
		p	0.737	0.019	0.049	0.624	<0.01		0.675	0.363
	Cu	r_s_	0.09	0.20	0.16	0.01	−0.18	0.08		0.02
		p	0.456	0.091	0.184	0.926	0.142	0.519		0.900
	Zn	r_s_	0.31	0.45	0.20	−0.02	0.13	−0.33	0.19	
		p	0.009	<0.01	0.091	0.898	0.297	0.004	0.119	

Phe: phenylalanine, As: arsenic, Cd: cadmium, Cu: copper, Hg: mercury, Mn: manganese, Pb: lead, Se: selenium, Zn: zinc.

## Data Availability

The data presented in this study are available on request from the corresponding authors due to privacy.

## Supplementary Materials

The following supporting information can be downloaded at: https://www.mdpi.com/article/10.3390/nu16203463/s1, Table S1: Relationship between participant characteristics and heavy metals.
