# Supplementary material for: Heavy Metal and Trace Element Status and Dietary Determinants in Children with Phenylketonuria
Source: Nutrients. 2024 Oct 12;16(20):3463. doi: 10.3390/nu16203463 (PMC11509891; doi:10.3390/nu16203463)
Supplement: Supplementary file 1 [file nutrients-16-03463-s001.zip › nutrients-3250669-supplementary.pdf]

Table S1. Relationship between participant characteristics and heavy metals.

	Pb*	*p*	As*	*p*	Cd*	*p*	Hg*	*p*
Phe-restricted diet								
Yes (n=34)	1.33 (1.06-1.99)	0.059	0.42 (0.33-0.48)	0.498	1.48 (1.25-1.65)	0.761	3.88 (3.56-4.54)	0.120
No (n=71)	1.66 (1.09-2.17)		0.42 (0.33-0.52)		1.42 (1.27-1.66)		3.65 (3.45-4.04)	
Sex								
Girl (n=56)	1.65 (1.19-2.12)	0.555	0.44 (0.33-0.51)	0.729	1.47 (1.30-1.66)	0.230	3.66 (3.42-4.18)	0.439
Boy (n=49)	1.46 (1.08-2.1)		0.41 (0.33-0.52)		1.41 (1.21-1.66)		3.84 (3.54-4.40)	
Age								
<4 years (n=59)	1.66 (1.08-2.21)	0.099	0.44 (0.35-0.54)	0.058	1.45 (1.26-1.67)	0.646	3.65 (3.39-4.27)	0.385
≥4 years (n=46)	1.38 (1.06-1.99)		0.41 (0.32-0.46)		1.44 (1.26-1.63)		3.86 (3.51-4.33)	
BAZ								
≤-1 (n=11)	1.46 (1.01-2.21)		0.44 (0.36-0.57)		1.42 (1.23-1.48)		3.56 (3.26-3.65)	
-1< <1 (n=64)	1.66 (1.15-2.13)	0.333	0.43 (0.34-0.52)	0.110	1.45 (1.25-1.64)	0.057	3.89 (3.51-4.36)	0.102
≥1 SDS (n=30)	1.22 (1.04-2.06)		0.39 (0.31-0.51)		1.58 (1.34-1.73)		3.67 (3.47-4.04)	
Diagnosis								
HPA (n=38)	1.67 (1.08-2.23)		0.42 (0.34-0.55)		1.44 (1.29-1.67)		3.75 (3.48-4.20)	
PKU (n=30)	1.33 (1.07-1.95)	0.146	0.40 (0.33-0.46)	0.516	1.51 (1.28-1.70)	0.638	3.88 (3.55-4.57)	0.317
BH4 responsive (n=37)	1.66 (1.09-2.13)		0.44 (0.33-0.52)		1.42 (1.23-1.64)		3.65 (3.43-4.04)	
Birth order								
First child (n=50)	1.56 (1.09-2.06)	0.805	0.42 (0.31-0.51)	0.211	1.46 (1.33-1.68)	0.240	3.86 (3.54-4.37)	0.214
1≥2. child (n=55)	1.64 (1.06-2.14)		0.44 (0.36-0.52)		1.42 (1.24-1.65)		3.65 (3.41-4.16)	
Total number of children								
1 (n=43)	1.65 (1.14-2.07)		0.42 (0.31-0.51)		1.46 (1.27-1.69)		3.95 (3.53-4.53)	
2 (n=38)	1.59 (1.01-2.09)	0.666	0.45 (0.33-0.56)	0.718	1.43 (1.25-1.64)	0.497	3.67 (3.47-4.02)	0.423
3 and more (n=24)	1.56 (1.07-2.16)		0.44 (0.36-0.51)		1.45 (1.30-1.66)		3.66 (3.28-4.35)	
Family structure								
Nuclear (n=89)	1.62 (1.10-2.11)	0.524	0.42 (0.33-0.51)	0.614	1.45 (1.29-1.67)	0.301	3.84 (3.47-4.30)	0.636
Extended (n=16)	1.59 (1.01-2.17)		0.42 (0.37-0.55)		1.40 (1.16-1.65)		3.65 (3.54-4.11)	
Perception of economic level								
Income is less than expenses (n=33)	1.66 (1.09-2.10)		0.44 (0.36-0.52)		1.45 (1.31-1.64)		3.65 (3.34-4.23)	
Income is equal to expenses (n=53)	1.41 (1.07-2.10)	0.330	0.39 (0.32-0.47)	0.062	1.42 (1.23-1.65)	0.107	3.69 (3.52-4.30)	0.605
Income is more than expenses (n=19)	1.67 (1.28-2.14)		0.48 (0.39-0.56)		1.62 (1.38-1.74)		3.88 (3.51-4.48)	
Total breastfeeding time, month								
0-5 (n=24)	1.56 (1.05-1.85)		0.45 (0.38-0.51)		1.57 (1.36-1.71)		3.97 (3.56-4.32)	
6-11 (n=22)	1.50 (1.11-2.12)	0.425	0.40 (0.35-0.48)	0.573	1.36 (1.25-1.47)	0.069	3.85 (3.55-4.60)	0.385
12-23 (n=29)	2.03 (1.13-2.21)		0.44 (0.33-0.57)		1.54 (1.29-1.66)		3.62 (3.31-4.51)	

24 and more (n=39)	1.64 (1.05-2.09)		0.42 (0.31-0.51)		1.47 (1.21-1.68)		3.65 (3.45-4.00)	
Maternal education level								
≤8 years (n=64)	1.56 (1.05-2.13)	0.458	0.42 (0.33-0.52)	0.589	1.45 (1.26-1.64)	0.346	3.67 (3.45-4.28)	0.875
>8 years (n=41)	1.67 (1.17-2.10)		0.45 (0.33-0.51)		1.47 (1.28-1.68)		3.76 (3.51-4.17)	
Paternal education level								
≤8 years (n=64)	1.56 (1.03-2.13)	0.503	0.42 (0.33-0.52)	0.793	1.45 (1.27-1.64)	0.349	3.68 (3.48-4.25)	0.730
>8 years (n=41)	1.64 (1.13-2.08)		0.42 (0.33-0.51)		1.46 (1.26-1.72)		3.68 (3.47-4.30)	
Parent's smoking status								
One or both of (n=65)	1.56 (1.08-2.07)	0.450	0.42 (0.33-0.52)	0.611	1.42 (1.27-1.67)	0.984	3.67 (3.48-4.30)	0.877
None of (n=40)	1.67 (1.12-2.20)		0.43 (0.33-0.51)		1.46 (1.26-1.65)		3.69 (3.48-4.25)	
Frozen food								
Yes (n=44)	1.50 (1.10-2.07)	0.671	0.42 (0.31-0.51)	0.245	1.47 (1.23-1.69)	0.514	3.68 (3.46-4.44)	0.652
No (n=61)	1.65 (1.05-2.15)		0.43 (0.34-0.54)		1.45 (1.31-1.64)		3.69 (3.49-4.11)	
Canned food								
Yes (n=57)	**1.67 (1.16-2.21)**	**0.016**	**0.46 (0.38-0.55)**	**0.004**	**1.56 (1.32-1.69)**	**0.007**	**3.88 (3.55-4.48)**	**0.028**
No (n=48)	**1.30 (1.04-2.02)**		**0.39 (0.33-0.45)**		**1.39 (1.22-1.58)**		**3.60 (3.28-4.07)**	
Canned beverage								
Yes (n=40)	1.30 (1.07-1.98)	0.178	0.40 (0.33-0.51)	0.607	1.54 (1.34-1.69)	0.110	3.89 (3.60-4.35)	0.096
No (n=65)	1.66 (1.14-2.16)		0.43 (0.33- 0.52)		1.41 (1.25-1.64)		3.64 (3.46-4.17)	
Buying a new furniture in the last year								
Yes (n=24)	1.66 (1.14-2.12)	0.633	0.44 (0.32-0.51)	0.725	1.56 (1.31-1.69)	0.219	**4.02 (3.69-4.58)**	**0.017**
No (n=81)	1.54 (1.07-2.11)		0.42 (0.33-0.52)		1.42 (1.26-1.64)		**3.65 (3.42-4.15)**	
Equipment for use in food preparation and/or consumption								
Glass								
Yes (n=47)	1.65 (1.09-2.13)	0.345	0.42 (0.36-0.52)	0.706	1.45 (1.26-1.65)	0.767	3.67 (3.51-4.16)	0.857
No (n=58)	1.56 (1.06-2.09)		0.42 (0.33-0.51)		1.45 (1.28-1.67)		3.86 (3.47-4.27)	
Porcelain/ceramics								
Yes (n=88)	1.65 (1.10-2.13)	0.210	**0.44 (0.34-0.52)**	**0.044**	1.45 (1.26-1.67)	0.689	3.77 (3.48-4.31)	0.240
No (n=17)	1.14 (1.00-1.99)		**0.36 (0.26-0.47)**		1.45 (1.31-1.63)		3.66 (3.38-3.95)	
Water used for cooking and/or drinking								
Tap water								
Yes (n=64)	1.60 (1.07-2.14)	0.916	0.42 (0.34-0.51)	0.579	1.47 (1.28-1.68)	0.181	3.67 (3.47-4.24)	0.745
No (n=41)	1.64 (1.12-2.06)		0.43 (0.30-0.52)		1.39 (1.23-1.64)		3.88 (3.52-4.33)	
Spring water								
Yes (n=14)	2.03 (1.07-2.25)	0.439	0.43 (0.35-0.58)	0.555	1.64 (1.35-1.74)	0.157	3.78 (3.49-4.70)	0.360
No (n=91)	1.57 (1.09-2.07)		0.42 (0.33-0.51)		1.42 (1.26-1.64)		3.68 (3.47-4.16)	
Bottled water								

Yes (n=39)	1.56 (1.11-2.09)	0.743	0.43 (0.33-0.51)	0.760	1.45 (1.26-1.66)	0.887	3.68 (3.51-4.28)	0.950
No (n=66)	1.66 (1.07-2.13)		0.42 (0.33-0.52)		1.45 (1.28-1.66)		3.68 (3.47-4.27)	
Purified water								
Yes (n=30)	1.66 (1.09-2.09)	0.766	0.38 (0.26-0.53)	0.307	**1.37 (1.17-1.52)**	**0.020**	3.88 (3.47-4.10)	0.927
No (n=75)	1.57 (1.08-2.13)		0.43 (0.36-0.51)		**1.53 (1.28-1.67)**		3.67 (3.48-4.28)	
Toys								
Painted wood								
Yes (n=44)	1.65 (1.10-2.12)	0.654	0.43 (0.33-0.51)	0.748	1.46 (1.30-1.69)	0.213	3.66 (3.46-4.04)	0.187
No (n=61)	1.57 (1.07-2.11)		0.41 (0.32-0.52)		1.45 (1.26-1.64)		3.84 (3.49-4.51)	
Unpainted wood								
Yes (n=59)	1.65 (1.08- 2.13)	0.396	0.43 (0.33-0.52)	0.567	1.41 (1.26-1.67)	0.465	3.64 (3.47-4.16)	0.346
No (n=46)	1.55 (1.06-2.07)		0.42 (0.33-0.51)		1.51 (1.33-1.65)		3.89 (3.54-4.36)	
Plush								
Yes (n=88)	1.63 (1.09-2.13)	0.317	0.44 (0.33-0.52)	0.278	1.46 (1.27-1.66)	0.476	3.68 (3.47-4.16)	0.486
No (n=17)	1.56 (0.99-2.03)		0.39 (0.34-0.44)		1.38 (1.23-1.66)		3.84 (3.50-4.60)	

Given as *median (IQR).

BAZ: BMI for age z-score, HPA: hyperphenylalaninemia (who blood phenylalanine levels within 2-6 mg/dL without any treatment), PKU: Phenylketonuria, BH4: Tetrahydrobiopterin, Phe: Phenylalanine, SDS: standard deviation score.

A p-value of < 0.05 was considered to be statistically significant.
